# Phenology determines the robustness of plant–pollinator networks

**DOI:** 10.1038/s41598-018-33265-6

**Published:** 2018-10-05

**Authors:** Rodrigo Ramos–Jiliberto, Pablo Moisset de Espanés, Mauricio Franco–Cisterna, Theodora Petanidou, Diego P. Vázquez

**Affiliations:** 1 GEMA Center for Genomics, Ecology & Environment, Universidad Mayor, Santiago, Chile; 2 Programas de Postgrado, Facultad de Ciencias, Pontificia Universidad Católica de Valparaíso, Valparaíso, Chile; 3 Centre for Biotechnology and Bioengineering–CeBiB, Universidad de Chile, Santiago, Chile; 4 Centro Nacional del Medio Ambiente. Universidad de Chile, Santiago, Chile; 5 Department of Geography, University of the Aegean, Mytilene, Greece; 6 Instituto Argentino de Investigaciones de las Zonas Aridas - CONICET, Mendoza, Argentina; 7 Freiburg Institute for Advanced Studies, University of Freiburg, Freiburg im Breisgau, Germany; 8 Facultad de Ciencias Exactas y Naturales, Universidad Nacional de Cuyo, Mendoza, Argentina

## Abstract

Plant–pollinator systems are essential for ecosystem functioning, which calls for an understanding of the determinants of their robustness to environmental threats. Previous studies considering such robustness have focused mostly on species’ connectivity properties, particularly their degree. We hypothesized that species’ phenological attributes are at least as important as degree as determinants of network robustness. To test this, we combined dynamic modeling, computer simulation and analysis of data from 12 plant–pollinator networks with detailed information of topology of interactions as well as species’ phenology of plant flowering and pollinator emergence. We found that phenological attributes are strong determinants of network robustness, a result consistent across the networks studied. Plant species persistence was most sensitive to increased larval mortality of pollinators that start earlier or finish later in the season. Pollinator persistence was especially sensitive to decreased visitation rates and increased larval mortality of specialists. Our findings suggest that seasonality of climatic events and anthropic impacts such as the release of pollutants is critical for the future integrity of terrestrial biodiversity.

## Introduction

Many types of ecological systems are threatened by environmental pressures. For example, shifts in temperature and precipitation, land use change, soil impoverishment, pollution, species loss, biological invasions and habitat loss severely affect global biodiversity. These pressures could be of particular concern when they threaten plant–pollinator systems, as these mutualistic interactions are essential for the functioning of ecosystems, both natural and agricultural.

Ecological communities can be depicted as networks of interacting species, in which adverse effects exerted on one species could in principle cascade on many other species, with consequences for community structure and dynamics. Of course, not all species contribute equally to maintaining community stability: species with particular topological attributes, such as high connectivity, are particularly important in this sense. For example, the loss of species with many connections to other species often impacts communities more strongly than the loss of species with few connections^1–3^. In the same vein, losing species that contribute more to nestedness in mutualistic networks leads to stronger impacts on community integrity^4^, while losing peripheral species (those with few connections within and between modules) in modular networks exerts only weak effects at the community level^5^. Fewer advances have been made towards understanding how stability of mutualistic networks is influenced by more inherent biological attributes related to functional traits, such as life cycle, body size, life form, invasiveness or phenology^6–8^. Arguably, to make progress in our understanding of the functioning of ecological networks we need an integrated evaluation of both purely topological attributes, which depend strongly on local community structure, and species’ biological attributes.

**Figure 1:**
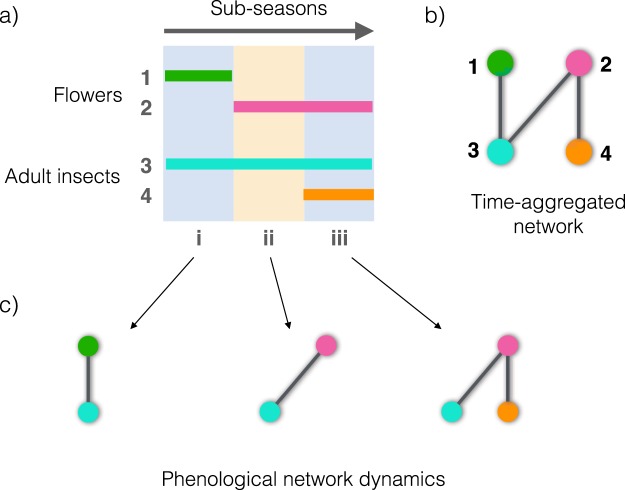
Graphical representation of the within-year temporal dynamics of networks driven by species phenologies. (**a**) Flowering phenology (species 1 and 2) and pollinator activity (species 3 and 4) of a hypothetical plant–pollinator web. Horizontal bars represent phenophases (activity periods) of each species throughout three consecutive sub-seasons (i, ii and iii). (**b**) Bipartite graph corresponding to the plant–pollinator network resulting from (**a**), aggregated through all sub-seasons, as done in most studies. (**c**) Sequence of three snapshots corresponding to the structure of the web in each sub-season. Note that the structure of the time-aggregated network (**b**) is never realized at any given time, resulting in a picture quite different from the static view of network topology. Also, the interactions between plants and pollinators are not necessarily transitive. This means that, if an interaction is active in later sub-seasons (e.g. the one between species 2 and 4 occurring in sub-season iii), it cannot propagate effects to others occurring in the past (e.g. between species 3 and 1 in sub-season i).

Plants and their pollinators in a community can interact only when their “active stages” (open flowers, active visitors) co-occur spatially and temporally^9–12^. Considering the need for co-occurrence is particularly relevant given that in many regions plant and pollinator species exhibit a strong, species-specific seasonalilty in the abundance of their active stages (phenology). Yet, most previous analyses of mutualistic networks have ignored such phenological dynamics^13–15^, (but see^16–19^), even though such dynamics determine the likelihood of interaction occurrence and thus the transfer of ecological effects through chains of direct interactions. Thus, clearly, ignoring phenological patterns in the study mutualistic networks could lead to incorrect conclusions about their dynamics (Fig. 1).

Because the active stages of species are not distributed homogeneously throughout the activity season, the impact of species on network structure and dynamics will depend on their temporal position. In most systems, few plant species flower and few animal species are active at the beginning and the end of the activity season, with an activity peak in the middle^20^. Therefore, if species whose phenologies concentrate either at the middle or at the extreme of the season are perturbed, the impact at the network level is not expected to be the same. Consequently, we infer that different phenological features of the species in a pollination network should dictate different network sensitivities to species perturbations.

In this study we analyze the role of species phenological attributes, such as initiation, end, and duration of the plant flowering and pollinator emergence phenophases, as determinants of network sensitivity to impacts on their constituent species. To this end, we used a combination of high quality empirical data, mathematical modeling and intensive computer simulations. We evaluated the hypothesis that phenological attributes of the species in mutualistic plant–pollinator networks are at least as important as the number of interactions per species (i.e. its “degree”) as determinants of network sensitivity to perturbations.

## Results

**Figure 2:**
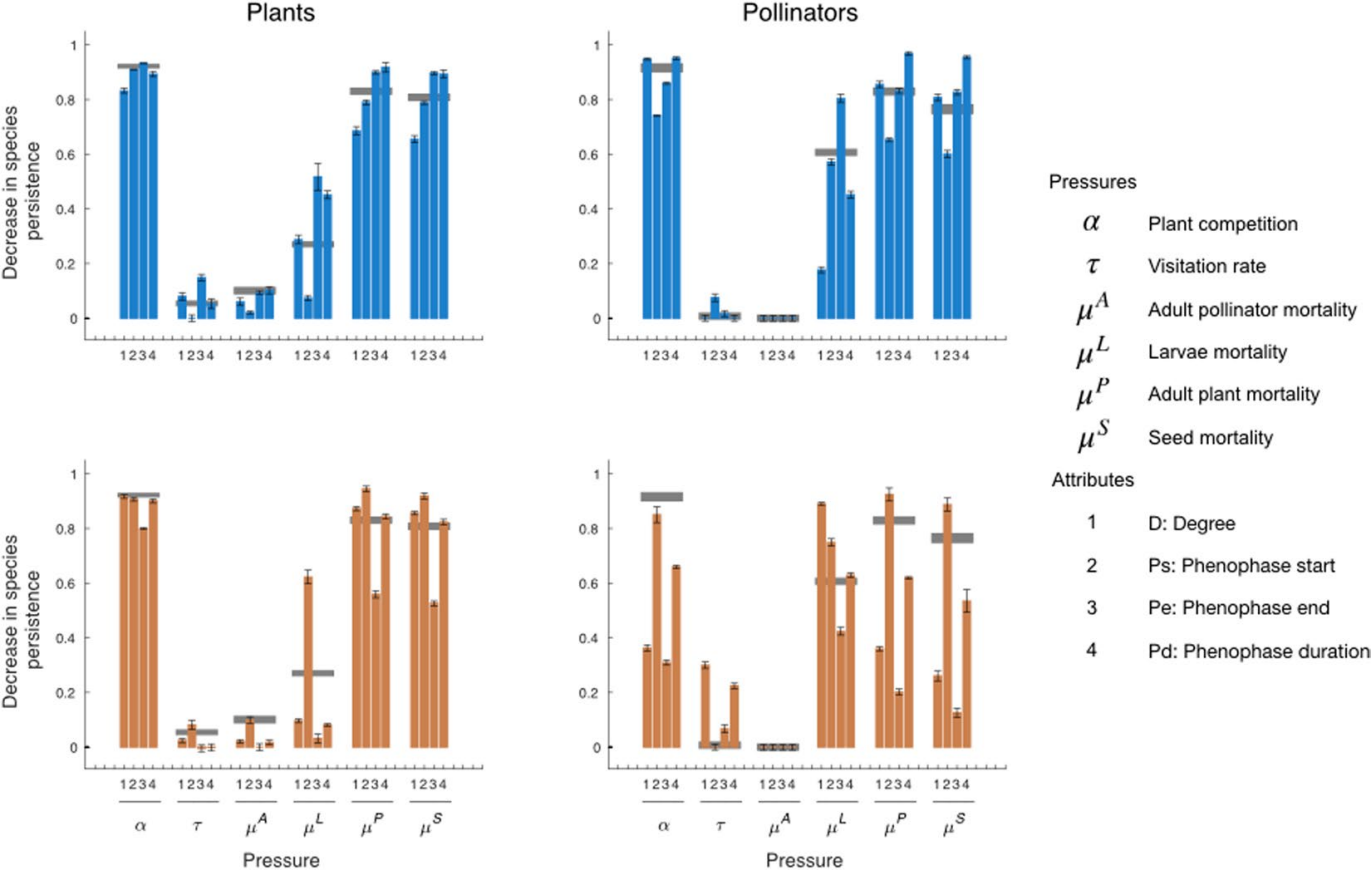
Simulation results for the Villavicencio (2011) plant–pollinator network (see Table 1). Left/right plots show decreases in plant/pollinator species persistence (mean ± 95% C.I.) after exerting different pressures, relative to species persistence without exerting any pressure (see text for details). Experimental pressures, applied one by one, were increased plant competition *α*, decreased visitation rate *τ*, and increased mortality of pollinators *μ*^*A*^, larvae *μ*^*L*^, adult plants *μ*^*P*^ and seeds *μ*^*S*^. Pressure intensities were assigned to species according to their attributes: 1: degree; 2: phenophase start; 3: phenophase end; 4: phenophase duration. Results for pressures assigned at random are shown as gray horizontal bars, whose heights indicate the 95% C.I. Blue bars: simulations in which pressure magnitude increased with increasing attribute values (low to high D, earlier to later Ps or Pe, and small to large Pd); orange bars: simulations for which pressure magnitude increased with decreasing attribute values.

For all plant–pollinator networks analyzed, changes in species persistence were driven mostly by pressures on plant competition intensity (*α*), pollinator visitation rate (*τ*), and mortality rates of seeds (*μ*^*S*^), adult plants (*μ*^*P*^), larvae (*μ*^*L*^) and adult insects (*μ*^*A*^; Supplementary Information S3). As results for all networks analyzed were qualitatively similar, we illustrate first the results only for the Villavicencio (2011) network, (Fig. 2), and later we provide a summary of the results for all networks.

As shown in Fig. 2, species persistence in the plant–pollinator network of Villavicencio Nature Reserve in 2011 was markedly affected by pressures exerted on plant competition (*α*), plant mortality (*μ*^*P*^ and *μ*^*S*^) and larval mortality of pollinators (*μ*^*L*^). However, the effects of intensifying plant competition is high regardless the attribute of species being attacked, Roughly speaking, no selective pressure was more damaging than random attacks. Plant persistence was affected especially when larval mortality (*μ*^*L*^) increased for pollinators with early phenophase initiation, late phenophase termination and longer phenophases. In turn, pollinator persistence was especially affected when larval mortality increased for pollinators with low degree, earlier phenophase initiation and late phenophase ending. Increased plant mortality (*μ*^*P*^), especially on plants with earlier and longer phenophases, led to decreased plant and pollinator persistence. Under increased seed mortality (*μ*^*S*^), the impact on both plant and pollinator persistence was stronger when the pressure was exerted on plants with earlier phenophase initiation, later phenophase termination and longer phenophases. Decreased visitation rate (*τ*) and adult pollinator mortality (*μ*^*A*^) had only weak effects on species persistence compared to other pressures. However, the observed effects of decreased visitation rate of pollinators with low degree or short phenophases were strong compared to decreasing visitation rates in species at random.

The conducted sensitivity analysis (see Supplementary Information S5) indicate that our results are robust to the choice of parameter values, as they remained similar with different levels of random variation in model parameter values.

**Figure 3:**
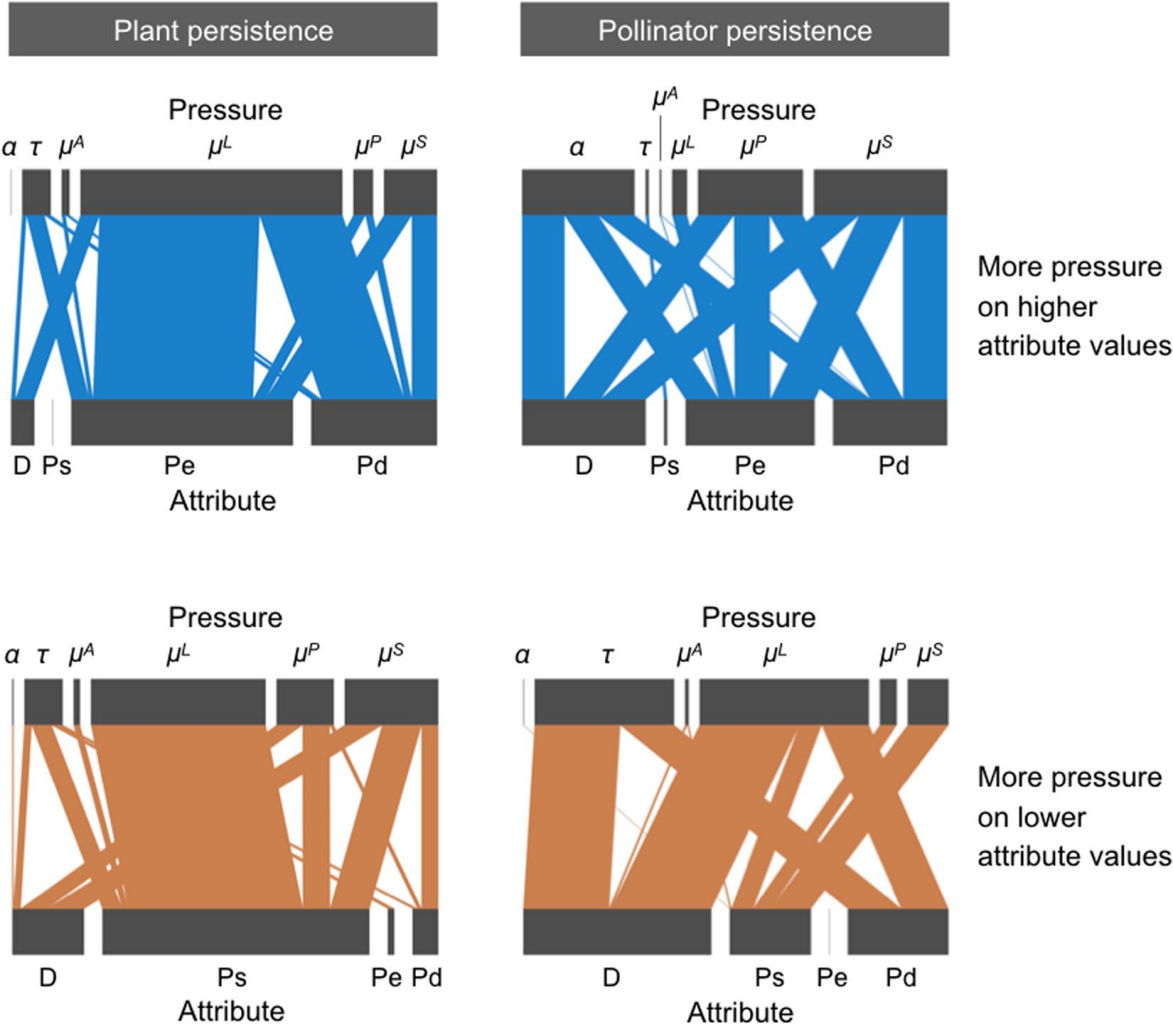
Summary of simulation results for all studied plant–pollinator networks described in Table 1. The webs summarize the adverse effects of different pressures (top blocks) exerted on species parameters (bottom blocks), according to values of species attributes. The width of the links are proportional to the corrected impact (see Methods, only positive values are shown). Perturbed parameters were plant competition intensity (*α*), pollinator visitation rate (*τ*), and mortality of adult pollinators, pollinator larvae, adult plants and seeds (*μ*^A^, *μ*^L^, *μ*^P^, *μ*^S^ respectively). Species attributes considered were degree (D), phenophase start (Ps), phenophase end (Pe) and phenophase duration (Pd). In top (blue) diagrams, pressure magnitude increased on species of increasing attribute values (e.g. low to high D, earlier to later Ps or Pe, and small to large Pd). In bottom (red) diagrams, pressure magnitude increased on species of decreasing attribute values. Left and right webs show respectively the resulting effects on plant and pollinator species persistence.

A general visualization of NMDS results can be seen in Supplementary Information S4. Mantel statistic was positive and significant (*r* = 0.4277; *P* < 0.01), indicating that results are more similar between networks of the same site. For this reason, we averaged the results among networks of the same site, and then averaged the (average) results of the four sites, to obtain the summarized outcome shown in Fig. 3. An integrated analysis of the 12 networks reveals that a subset of all attribute-pressure had a distinctively higher effect on species persistence (Fig. 3; see details in Supplementary Information S6). More precisely, plant persistence was affected the strongest by decreasing larval mortality rates in pollinators that exhibited earlier phenophase initiation or later phenophase termination. By contrast, pollinator species persistence was most affected by decreasing visitation rates and larval mortality in pollinators with low degree (i.e. pollinators that visit only few plant species). Overall, plant persistence was mainly affected when pressure was applied on pollinators (*μ*^L^). The effect of increasing this parameter is the highest when punishing pollinator species with longer phenophase, later phenophase end or earlier phenophase start. By contrast, Pollinator persistence was affected the most when specialist pollinator species (low *D*) were punished by either lowering the visitation rate *τ* or increasing the larvae mortality (*μ*^L^).

## Discussion

In this study we simulated generic pressures exerted on selected parameters of the species. These parameters represent mortality rates of both latent and active stages of plants and pollinators, visitation rates of pollinators on plants and competition among plants. These population-level parameters were selected as being prone to be altered by the major environmental pressures affecting pollination systems, as described below. We analyzed how the dynamics of the networks depended on the kind of species being perturbed, based of their phenological attributes and connectivity. Our analyses indicate that phenological attributes of plants and pollinators are strong determinants of network robustness, a result consistent across the networks we studied. Plant persistence was particularly sensitive to increased larval mortality of pollinators whose activity period starts early or finish late in the flowering/activity season. By contrast, pollinator persistence was most sensitive to decreased visitation rates and increased larval mortality of specialists. These results were consistent across the 12 pollination networks studied from four localities spanning diverse latitudes, elevations and climatic regions across the globe. Our results underscore the importance of considering the interplay between species’ phenological and life history attributes to understand the network-wide dynamics of plant–pollinator assemblages.

Our main findings suggest that environmental perturbations occurring at the beginning and at the end of each season are specially threatening to species persistence and community integrity. For example, increased occurrence of frosts at the beginning of the growing season or heat waves at the end of the growing season could affect species’ demographies and inter-specific interactions^21,22^. Impacts on pollinators, and specifically on larval survival and behavioral rhythms governing visitation rates are of special concern. Since network sensitivity to larval mortality is likely to show a seasonal trend with peaks at the extremes of the season, evaluating how the intensities of different pressures are spread over the flowering season could be useful for a correct assessment of community risks. In terms of risk analysis, the mutualistic network is likely to lose many species if perturbations occur during the extremes of the season, but, how likely is it for a perturbation to occur at those periods? How are disturbances distributed over time and how likely is it that they could overlap with the major pressures impacting pollinator performance? It is well known that several environmental pressures, such as pathogen incidence, predation risk and pollution, often exhibit strong seasonality^23–28^. Due to the changes expected in seasonal patterns of species and ecosystem functioning as a consequence of climate change^29^, it is uncertain how the peaks of environmental pressures will be displaced within the year. The potential increase in dose and frequency of pesticides application in the future, as a consequence of the changes in precipitation and temperature patterns^23,28^ is of particular concern.

Our study is a contribution for understanding and predicting some potential consequences of human-driven environmental change. In particular, human activities are leading to a global decline of many pollinator species^30^, which is a cause of concern given that the vast majority of angiosperm species depends on animal pollination^31^. This process threatens terrestrial biodiversity along with the functioning of ecosystems and the provision of services essential for human wellbeing. The major drivers of pollinator decline include climate change, landscape alteration and pollution, change in land use, decreased resource availability and diversity, biological invasions and increased pathogen transmission^30,32^. These drivers often act simultaneously and synergistically, which exacerbates their impact; for instance, biological invasions may be fostered by habitat alteration, while pathogen incidence is promoted by the introduction of alien species^32,33^. These pressures generate diverse impacts either on pollinators, on the plants they visit, or on the frequency and success of visitation events. For example, shifts in the mean and variance of ambient temperature may affect production of floral rewards for pollinators^34^, influencing their birth and death rates. In addition, numerous physiological effects on insects have been reported, including changes in activity rhythms—and hence visitation rates—, body size and life span. Also, flower production can decrease in response to warming, affecting pollinator visitation rates and the reproductive output of pollinators^34^. Landscape alteration, which involves multiple changes including land use, habitat fragmentation, and diffuse pollution, may reduce habitat availability and quality, pollinator abundance, visitation rates and plant pollination worldwide^30,35–37^. Decreasing diet diversity of pollinators, as a consequence of reduced native plant richness, depleted survival via reducing immunocompetence^38^. At the same time, several parasite species may attack pollinators, causing disease, death and population declines^30,39^. In the same vein, plant populations suffer from seed predation and parasitism^40^. Finally, regarding invasive species, some of their effects involve increasing competition among plants and among pollinators^33^ and decreasing visitation rates, especially in response to pollinator invaders^37^.

Our finding of the importance of species attributes for network dynamics and stability sets our work apart from what has been done before. Previous studies have revealed that topological attributes of species, particularly degree centrality (number of interactions that a species maintain with other species in the network), are strong determinants of the sensitivity of ecological networks to impacts exerted on those species^1–3^. These studies simulated species removals and measured the consequences at the network level in terms of species persistence, after determining secondary extinctions using simple static models. Static models have both merits and limitations derived from their simplicity. Although they omit several aspects of reality, have the strength of relying on only a small set of assumptions. At the other extreme, nonlinear dynamic models capture many elements considered essential from a biological point of view, but incorporate a considerable number of functions and parameters that are largely unknown. In the case of plant-pollinator networks, several studies have incorporated population dynamics^7,41,42^ but assuming that per capita interaction strengths are governed only by population sizes. More recently, a limited number of studies have considered interaction strengths as being time–varying. This is due to behavioral adaptation^43,44^ and/or to switching in interaction partners, also known as interaction rewiring^45,46^. These studies reveal that flexibility in both interaction strength and in the connections to other species^47^ strongly increases robustness of networks to environmental pressures^45,46^. Overall, results suggest that when species of high degree are removed from the network, the benefits of rewiring are stronger. Thus, regardless the approach (from static to dynamic with interaction rewiring), connectivity of species, and particularly species degree, has been used as the main predictor variable. Here, we adopted a nonlinear dynamic approach for revealing dynamic system responses to selective attacks on their constituent species. Our modeling approach allowed us to address our inquiry at two complementary time–scales: a short-term (i.e. phenological) scale and a long–term (asymptotic) one. The responses at the network level were analyzed considering the interplay between the attributes of the species and the type of pressure exerted on them.

In our modeling exercise we have manipulated what we judged were key species attributes governing network dynamics. However, other model parameters are suitable for analysis and relevant from an ecological point of view. For example, alteration of maturation rates of dormant states into adults may cause shifts in population structure and weakening of mutualistic interactions^48^. Such life-historical shifts may be driven by pollutant exposure such as neonicotinoids^49,50^. Other interesting candidates for analysis are those parameters related to production and consumption of floral rewards and competition for space among larvae and competition for flowers among active pollinators, in the frame of invasive species in mutualistic systems^44^. These issues deserve special attention and may be addressed in future studies using our model or similar ones.

Note that in creating our model, we took a mechanistic view of plant-pollinator interactions. This resulted in a mathematical asymmetry between growth equations of plants and pollinators, consequence of the biological asymmetry between them. This explains why the results are not interchangeable between plants and insects as it happens in more abstract studies of mutualistic networks^51^. There are plausible explanations for some of the results. Decreases in visitation rates (*τ*) hurt larvae population as larvae production depends on food intake which depends on how often an insect lands on a flower. In the particular case of specialist insects we observe that they usually visit generalist plants, because networks are typically nested^52^. If those pollinators are driven to extinction by a decrease of *τ*, the visited plants tend not to be much affected as other insects can pollinate them. A somewhat similar effect happens when increasing larvae mortality (*μ*^*L*^). This pressure has a straightforward effect on larvae abundance, which in turn reduces the number of free adults, even to the point of extinction. Considering that in empirical networks most pollinators are specialists^52^, this explains why applying pressure on insects with low degree is so negative for their persistence. Since the specialist insects are the ones prone to extinction, the same reasoning based on the nested structure of plant-pollinator networks presented above applies here, with most plants being unaffected. These explanations are consistent with the results presented in Fig. 3. The mechanisms that leads to plant extinctions are quite different. Plant persistence was mainly reduced when increasing mortality of pollinators that emerge at the extremes of the season. A preliminary analysis of our plant-pollinator networks shows that both early–emerging and late–emerging pollinators tend to have a high number of interactions. Thus, reducing abundance of them mostly affects specialist plants. These plants species, because of nestedness, are more common and depend strongly on generalist pollinators. This interplay between phenological and topological attributes calls for considering possible correlations among traits in future studies to build solid explanations of the collective behavior of pollination networks. Highly correlated species attributes may lead to artifacts, which should call for caution when interpreting our results. For example, species with long phenophases also tend to start earlier and finish later. As our results indicate, plant–pollinator networks are especially sensitive to perturbations on species exhibiting earlier and later phenophases. Thus, species of longer phenophases become also important because they tend to be active at the extremes of the season. In this sense, high correlation in the species attributes studied here could exacerbate the effects observed in our study, and our assumption of no correlation among species attributes makes our results more conservative.

From the point of view of conducting mutualistic interactions only adult pollinators are the active stages. However, our findings show that larval stages may have a preeminent importance for the long–term integrity of the community. Then, future studies could benefit from assessing the abundance of larval stages of pollinators, a sort of resting–stage bank, as an early signal of community risk. We guess that, as a next phase in mutualistic network research, topological data should be enriched with key demographical information. From this, new insights could be obtained to strengthen ecological theory and to foster the design of management and conservation actions.

As it is increasingly being recognized, the structure of species interactions is highly dynamic^18,53^. Our study highlights the fact that the traditional view of ecological networks as aggregated entities is incomplete in this regard. In particular, aggregated networks neglect the temporal shifts in structure, stability and robustness to environmental pressures. Considering species phenologies, the aggregated approach implicitly assume that phenologies extend over the whole season. However, the heterogeneity of phenophase properties allows to view a single network as a sequence of sub-networks with striking differences in species composition and interactions. This is true for intra-season^18^ as well as for inter-annual timescales^53^. This differences in structure are expected to drive differences in dynamics. As suggested by our study, the diversity of temporal phenological attributes of species strongly influences the predicted responses of ecological systems to environmental threats.

## Methods

### Dataset

We conducted this study on plant–pollinator networks from four locations. Each network included a set of species of flowering plants and their insect visitors, occurrence of mutualistic interactions (effective visits) between plant and animal species, and phenophases (initial and ending dates) of flowering and emergence of active pollinators. A summary of basic information about these empirical networks is presented in Table 1. At each location, two (Zackenberg), three (Athens), one (Nahuel Huapi), and six (Villavicencio) one-season subnetworks were obtained. For simplicity, we will refer to each of the 12 subnetworks as “network.”

**Table 1:** Basic information of the empirical networks used in this study.

Location	Habitat type	Sampling years	Network size	References
Zackenberg, Greenland. 74°30′N, 21°00′W; 35 m.a.s.l.	Heathland, old riverbank and snow beds	1996–1997	P = 31 A = 61	^20^
Athens, Greece. 38°00′N, 23°38′E; 175 m.a.s.l.	Mediterranean low scrub	1984–1986	P = 130 A = 592	^54^
Nahuel Huapi National Park, Río Negro and Neuquén Provinces, Argentina. 41°00′S, 71°30′W; 750–800 m.a.s.l.	Temperate forest	1999	P = 17 A = 146	^55, 56^
Villavicencio Nature Reserve, Mendoza, Argentina. 32°31′S, 68°56′W; 1270 m.a.s.l.	Dry scrubland	2006–2011	P = 59 A = 187	^10, 57^

P/A indicates number of plant/animal species in the network.

### The dynamic model

**Figure 4:**
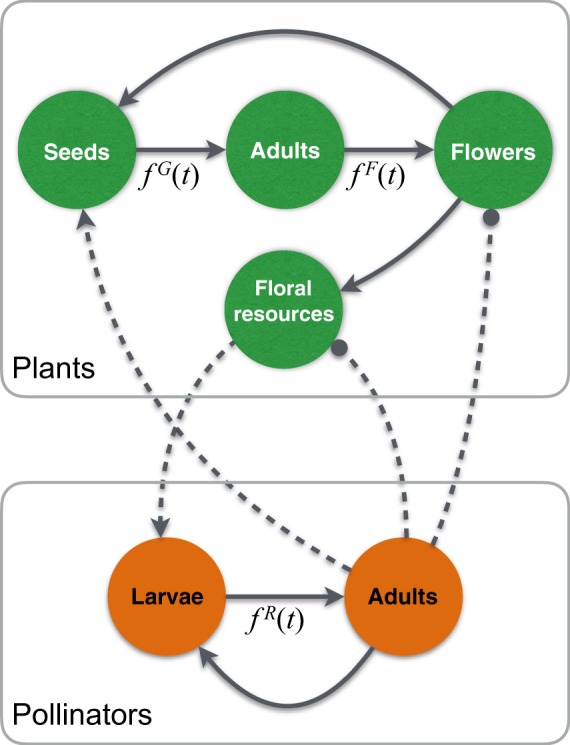
Graphical representation of the basic structure of our dynamic model. Lines ended in arrows/circles indicate a positive/negative effect of one variable on another. Continuous lines indicate life-cycle transitions within a species. Dashed lines represent interactions between plants and their pollinators. Positive effects compose the classically depicted mutualistic interaction. Negative effects arise by pollinators’ flower exploitation (see text for details). Self-effects not shown to keep simplicity. Those transitions marked with *f*(*t*) are governed by time-dependent (i.e. phenological) functions. In these functions, superscripts *g*, *f* and *r* represent germination, flowering and pollinator recruitment, respectively.

We built a dynamic model for networks of plant–pollinator interactions. The model involves a set of non-autonomous integro-differential equations in partial derivatives. Plants were modeled by four state variables: seeds, adult plants, flowers and floral resources. Pollinators are considered to be only insects for simplicity, and were structured into larvae and adults. The basic structure of our dynamic model is shown in Fig. 4, and the details are provided in Supplementary Information S1 and S2. A key feature of our model is that seed germination, flowering and insect recruitment are phenological events governed by time-dependent smooth–pulse (Gaussian) functions, here called phenology functions. These functions represent the environmental control of phenological events, in the sense that they could occur only when *f*(*t*) > 0. The time interval during which phenology functions are activated was adjusted so that the species phenophases exhibited by model simulations matched empirical phenophases. Another key feature of our model is that recruitment of dormant states of plants (seeds) and animals (larvae) also obey an internal control. This internal control means that dormants must surpass a given age functions to recruit. Thus, while all state variables depend on time, seeds and larvae depend also on age.

### Experimental design

We applied the following pressures (one by one) to the model parameters of every species of plant or pollinator: Decreasing plant carrying capacity, decreasing visitation rate, increasing mortality of adult insects, increasing mortality of larvae, increasing mortality of adult plants and increasing seed mortality.

We evaluated the role of phenological traits of species, as compared to their topological properties, as determinants of network robustness to environmental pressures. To this end, we applied each pressure to the species according to the values of some phenological and topological attributes empirically recorded. The phenological attributes studied were starting time of the phenophase, ending date of the phenophase and duration (difference between ending and starting time) of the phenophase. The only topological attribute of the species we considered was degree (the number of interactions with other species in the network). We sorted the species in the network according to each of their measured attributes and applied each pressure at increasing and decreasing intensity. For example, when we studied the role of species degree, we applied first less pressure to low-degree species and more pressure to high-degree species, in a linear way. Then we reversed the treatment and applied more pressure to low-degree species and less pressure to high-degree species. The same procedure was executed for each of the six pressure types and the four species attributes plus random sorting, both with increasing and decreasing pressure intensity. We run 15 replicates for each treatment, which resulted in 9,000 model runs conducted independently on each of the 12 empirical networks utilized.

### Simulation

Each simulation was run for 6,000 time steps of one week each without any pressure to discard transient dynamics. At the end of this phase, all species trajectories reached a characteristic behavior in their biomass, driven by the time-dependent phenologies. This behavior is analogous to the asymptotic steady state in classic autonomous systems. After this transient phase, the simulations were run for additional 6,000 time steps, which correspond to the experimental phase in which the different treatments were applied. We used the integration algorithm ode23 within MATLAB release 2016a (The Mathworks Inc., Natick, Massachusetts).

All parameter values were drawn from a uniform random distribution centered at their basal values (see Supplementary Information S2) ±25%. In addition, we performed a sensitivity analysis on one of the networks to check the robustness of our results to different levels of random variation in parameter values. Specifically, we compared our results after varying model parameters in 10, 25, 50 and 75% from their basal values. Basal parameter values were obtained from realistic estimates or based on empirical measures reported in literature when available. Initial conditions used in our simulations are shown in Supplementary Table S2.

### Pressure intensities

We simulated a sustained pressure on species over *Y* years by changing the values of some of their parameters. Consider the value of any parameter *x*_*i*, *T*_ for species *i* during year *T*. We updated it as:

$${x}_{i,T+1}={x}_{i,T}\cdot {\hat{z}}_{i}$$

We call *x*_*i*,0_ the basal value of the target parameter (see Table S1). ${\hat{z}}_{i}$ represents the pressure intensity exerted on a target parameter of species *i*. Notice that we can represent increases in mortality rates or competition by choosing ${\hat{z}}_{i}$ > 1 and decreases in visitation rates with ${\hat{z}}_{i}$ < 1. The values of ${\hat{z}}_{i}$ are drawn from a uniform random distribution centered at $\sqrt[Y]{{z}_{i}}\pm \mathrm{5 \% }$, with *z*_*i*_ defined as follows: Each species suffered a different pressure intensity, according to the value of its attribute of interest. For example, if we study the role of species degree for network robustness, we first sorted the species from low to high degree, generating a list Σ. Species of the same degree were included only once. Then, we associated to that list a vector of time-aggregated pressure intensities ***Z*** = [1... *z*_*max*_] of the same length as Σ and with element values equally spaced. The values of maximal aggregated pressure *z*_*max*_ were equal to 50 for all mortality rates (seeds, adult plants, larvae and active pollinators), 50 for plant competition and 1/50 for visitation rate. A species was considered extinct when its biomass fell to zero in all their states-variables. As a measure of network robustness, we recorded species persistence as the number of species with positive biomass at the end of the simulation over the number of species with positive biomass at the end of the transient phase.

At the end of the Results section we show an analysis combining data for all studied networks. We focus, for each specific pressure, on the difference between the reduction in species persistence after affecting preferentially species with certain attribute values (e.g. species with lower degree, earlier phenophase initiation, etc.) and the reduction in species persistence after affecting species at random (i.e., independently of attribute values). The magnitude of this difference is referred to as “corrected impact” on the network. To evaluate differences in our results (corrected impact driven by the different pressures) among networks, we performed a non-metric multidimensional scaling (NMDS) analysis. We also tested, with a Mantel test, the null hypothesis that the corrected impacts on plant and pollinator persistence differ between networks of different sites but do not differ between networks of different years in the same site.

## Electronic supplementary material

Supplementary information
