# Supplementary material for: Phenology determines the robustness of plant–pollinator networks
Source: Sci Rep. 2018 Oct 5;8:14873. doi: 10.1038/s41598-018-33265-6 (PMC6173761; doi:10.1038/s41598-018-33265-6)
Supplement: Supplementary file 1 — Supplementary information [file 41598_2018_33265_MOESM1_ESM.pdf]

Supplementary information

for

**Phenology determines the robustness of plant--pollinator networks**

Rodrigo Ramos—Jiliberto, Pablo Moisset de Espanés, Mauricio Franco—Cisterna, Theodora Petanidou, Diego P. Vázquez

## Supplementary Information S1: Model details

The model we developed governs the biomass dynamics of a complex interaction system of flowering plants and their insect pollinators. Each plant species is composed of four state-variables: (1) Adult plants, able to produce flowers and seeds, (2) flowers, that can be visited by pollinators to be fertilized, (3) floral rewards, which are used by pollinators as food, and (4) seeds, produced by adult plants and assumed to be obligate dormants.

For simplicity, all flower-visiting insects are considered as pollinators. They are structured into two developmental states: (1) Adult insects, able to visit flowers, consume floral rewards and produce larvae, and (2) Larvae, a single juvenile stage assumed to be the dormant state of pollinators. For all states, biomass density ($g/m^2$) is used as the unit.

Transition from a dormant state, either seed or larva, to the adult state occur when two conditions are simultaneously met: (a) a given latency period has finished, implying that the dormants reached a minimum age, and (b) environmental conditions are favorable for seed germination or insect recruitment, which is dictated by time-dependent functions $f^G(t)$ and $f^R(t)$ respectively (see Fig.4).

Adult plants can produce flowers in a given period of the year, and flowers produce floral rewards for pollinators. The phenological flowering dynamics is governed by the time-dependent function $f^F(t)$. During flowering, adult insects visit flowers and consume floral rewards. As a consequence of visits, some flowers are fertilized, getting unavailable for further interactions, pollinators obtain food that will be converted into offspring and plants reproduce giving rise to dormant seeds.

The mathematical model proposed is a system of integro-differential equations on partial derivatives. Phenophases of germination, flowering and insect recruitment are described by time-dependent functions, named here "phenology functions" ($f(t)$), that describe the within-year time evolution of the level of environmental favorability for each of the phenological events. All the state-variable values depend on chronological time ($t$). For dormants (seeds and larvae), state-variable values depend on age ($u$) too.

Variation on seeds biomass is given by :

$$\frac{\partial S_i(t,u)}{\partial t} = -\frac{\partial S_i(t,u)}{\partial u} - \mu_i^S S_i - \sigma_i S_i f_i^g(t) g_i^S(u) \tag{1}$$

where the first term on right side is an advection term, which describes loss of seeds of age $u$ due to aging. The second term is seed mortality and the last term represents biomass loss due to recruitment from dormant to adult stage. This term is composed of parameter $\sigma_i$, representing the rate at which seeds transit to adult plants, environmental favorability for germination $f_i^G(t)$, and the maturation function ($g(u)$) that describes the biological readiness for germination as a function of seed age:

$$g(u)_i^S = \begin{cases} 1 & \text{if } u \geq u_0 \\ 0 & \text{otherwise} \end{cases}, \tag{2}$$

with $u_0$ being an age threshold above which seeds are mature and ready to germinate. Seed production is given by:

$$S_i(t,0) = \sum_j \pi_{ij} \varphi_{ij}(A_j, F_i) \tag{3}$$

and is proportional to the visitation rate ($\varphi_{ij}$) to flowers of plant species $i$ ($F_i$) by pollinators of specie $j$ ($A_j$). This function was modeled as a Beddington-DeAngelis-like functional response:

$$\varphi(A_j, F_i) = \left( \frac{\tau_{ij} A_j F_i}{\beta_{ij} + a_{ij} \sum_{k \in R(j)} F_k + b_{ij} \sum_{l \in C(i)} A_l} \right) \tag{4}$$

where the set $R(j)$ are plant species visited by pollinator $j$ and the set $C(i)$ are pollinator species that visit plant $i$.

Biomass dynamics of adults plants are given by:

$$\frac{dP_i(t)}{dt} = \left( 1 - \alpha_{ii} P_i - \sum_{k \neq i} \alpha_{ik} P_k \right) v_i \int_0^\infty \sigma_i f_i^g(t) g_i^S(u) S_i du - \mu_i^P P_i \tag{5}$$

Plant biomass growth, due to seed germination, is limited by intra- and inter-specific competition, which is regulated by parameters $\alpha_i$ and $\alpha_k$. The last term is mortality rate of plants.

Biomass dynamics of flowers are described by:

$$\frac{dF_i(t)}{dt} = r_i^F\left(\kappa_i P_i - F_i\right) f_i^f(t) - \left(\mu_i^F + \rho_i^F\left(1 - f_i^f(t)\right)^2\right) F_i - \sum_j \gamma_{ij}\varphi_{ij}(A_j, F_i) \tag{6}$$

where the first term represents limited flower growth sustained by plant biomass, and favored by the phenology function $f_i^F$. The second term represent time-dependent mortality, composed by a basal mortality rate $\mu_i^F$ and a seasonally-driven extra mortality that is maximal, with value $\rho_i^F$ outside the flowering phenology period dictated by $f(t)$. The third term is a removal of fertile flower biomass due to visitation-driven fertilization.

The dynamic equation for floral resources is:

$$\frac{dN_i(t)}{dt} = r_i^N(q_i F_i - N_i) - \sum_j \delta_{ij}\varphi_{ij}(A_j, F_i)\left(\frac{\frac{N_i}{F_i}}{\varepsilon_{ij} + \frac{N_i}{F_i}}\right) \tag{7}$$

which has a term of limited growth sustained by flower biomass, and a term of biomass loss due to consumption by pollinators.

Biomass growth rate of insects is given by:

$$\frac{\partial L_j(t,u)}{\partial t} = -\frac{\partial L_j(t,u)}{\partial u} - \mu_j^L L_j - \chi_j L_j f_j^r(t) g_j^L(u) \tag{8}$$

This equation has a structure equivalent to the equation of seeds (1). Production rate of larvae is described by the boundary condition:

$$L_j(t,0) = \left(1 - \omega_{jj}L_j - \sum_{k\neq j}\omega_{jk}L_k\right)\sum_i \eta_{ij}\delta_{ij}\varphi_{ij}(A_j, F_i)\left(\frac{\frac{N_i}{F_i}}{\varepsilon_{ij} + \frac{N_i}{F_i}}\right) \tag{9}$$

Production of larvae depends on consumption of floral resources by adult insects, and it is limited by intra- and inter-specific competition, governed by $\omega_i$ and $\omega_j$.

Finally, biomass density growth rate of adults insects is:

$$\frac{dA_j(t)}{dt} = y_j \int_0^\infty \chi_j f_j^r(t) g_j^L(u) L_j du - \left(\mu_j^A + \rho_j^A\left(1 - f_j^r(t)\right)^2\right) A_j \tag{10}$$

The population growth rate of insects is governed by recruitment, in an equivalent form than for plants (eqn. 5). Insect mortality has, similar to eqn. 6, a basal mortality term $\mu_j^A$ and a seasonal mortality with maximum $\rho_j^A$ outside the recruitment phase dictated by $f_j^R$.

All parameter values were drawn at random from uniform probability distributions, centered at estimated mean values (Table ) $\pm 25\%$. Initial estimation of mean parameter values was based on biological plausibility and in some cases guided by literature data available for some species. After that, mean values were adjusted as to avoid very low species persistence.

## Supplementary Information S2: Model parameters

Table S1: Model parameters: symbols, short description, mean values. Dimensionless parameters are shown as "dl"

| Symbol | Description | Value | Unit |
|---|---|---|---|
| $\mu^S$ | Basal seed mortality | $1.3 \cdot 10^{-2}$ | $week^{-1}$ |
| $\mu^P$ | Basal plant mortality | $2.8 \cdot 10^{-3}$ | $week^{-1}$ |
| $\mu^F$ | Basal flower mortality | $8.8 \cdot 10^{-1}$ | $week^{-1}$ |
| $\mu^A$ | Basal adult insect mortality | $4.4 \cdot 10^{-2}$ | $week^{-1}$ |
| $\mu^L$ | Basal larva mortality | $1.0 \cdot 10^{-2}$ | $week^{-1}$ |
| $\rho^F$ | Maximal winter extra mortality of flowers | 10.0 | $week^{-1}$ |
| $\rho^A$ | Maximal winter extra mortality of insects | 10.0 | $week^{-1}$ |
| $r^F$ | Production rate of flowers by unit plant biomass | 2.0 | $week^{-1}$ |
| $r^N$ | Production rate of floral resources by unit flower biomass | 4.2 | $week^{-1}$ |
| $\nu$ | Conversion rate from seeds to plants | $1.0 \cdot 10^{3}$ | dl |
| $y$ | Conversion rate from larvae to adult insect | $4 \cdot 10^{-1}$ | dl |
| $\sigma$ | Germination rate of mature seeds | $1.1 \cdot 10^{-1}$ | $week^{-1}$ |
| $\chi$ | Recruitment rate of mature larvae | 1.0 | $week^{-1}$ |
| $\kappa$ | Flower carrying capacity per unit plant biomass | $2 \cdot 10^{-2}$ | dl |
| $q$ | Floral resources carrying capacity per unit flower biomass | $1 \cdot 10^{-1}$ | dl |
| $\tau$ | Maximum visitation rate per unit insect biomass | $1 \cdot 10^{6}$ | $g^{-1}\ m^2\ week^{-1}$ |
| $\delta$ | Resource consumption per visit | $1.5 \cdot 10^{-2}$ | dl |
| $\gamma$ | Flower biomass loss per visit | $7 \cdot 10^{-3}$ | dl |
| $\pi$ | Maximum seed production per visit | $1.2 \cdot 10^{-1}$ | dl |
| $\eta$ | Conversion efficiency of floral resources to larvae production | $4.1 \cdot 10^{-1}$ | dl |
| $\beta$ | Half-saturation parameter of visitation rate function | 1.0 | dl |
| $\varepsilon$ | Half-saturation parameter of per-visit consumption function | $2.5 \cdot 10^{-2}$ | dl |
| $a$ | Coefficient of competition among flowers for insect visitation | $1.0 \cdot 10^{-3}$ | $g^{-1}\ m^2$ |
| $b$ | Coefficient of competition among insects for visiting flowers | $1 \cdot 10^{2}$ | $g^{-1}\ m^2$ |
| $\alpha_{ii}$ | Coefficient of intra-specific competition among plants for space | $1 \cdot 10^{-3}$ | $g^{-1}\ m^2$ |
| $\alpha_{ij}$ | Coefficient of inter-specific competition among plants for space | $1 \cdot 10^{-4}$ | $g^{-1}\ m^2$ |
| $\omega_{ii}$ | Coefficient of intra-specific competition among insect larvae for space | $5 \cdot 10^{2}$ | $g^{-1}\ m^2$ |
| $\omega_{ij}$ | Coefficient of inter-specific competition among insect larvae for space | $5 \cdot 10^{1}$ | $g^{-1}\ m^2$ |

Table S2: Initial and boundary conditions of state variables. For each species, we divided the shown value by the number of species in its group.

| Symbol | Description | Value | Unit | Extinction threshold |
|---|---|---|---|---|
| $P_i(0)$ | Adult plants | $9.9 \cdot 10^1$ | $g\ m^{-2}$ | $1 \cdot 10^{-3}$ |
| $F_i(0)$ | Flowers | 2.0 | $g\ m^{-2}$ | 0 |
| $N_i(0)$ | Floral resources | $3 \cdot 10^{-2}$ | $g\ m^{-2}$ | 0 |
| $A_i(0)$ | Adult insects | $3 \cdot 10^{-3}$ | $g\ m^{-2}$ | $1 \cdot 10^{-8}$ |
| $S_i(0,u)$ | Seeds of age $u$ | 6.8 | $g\ m^{-2}\ week^{-1}$ | $1 \cdot 10^{-5}$ |
| $L_i(0,u)$ | Insect larvae of age $u$ | $7 \cdot 10^{-4}$ | $g\ m^{-2}\ week^{-1}$ | $1 \cdot 10^{-8}$ |

## Supplementary Information S3: Simulation results for all tested plant-pollinator networks

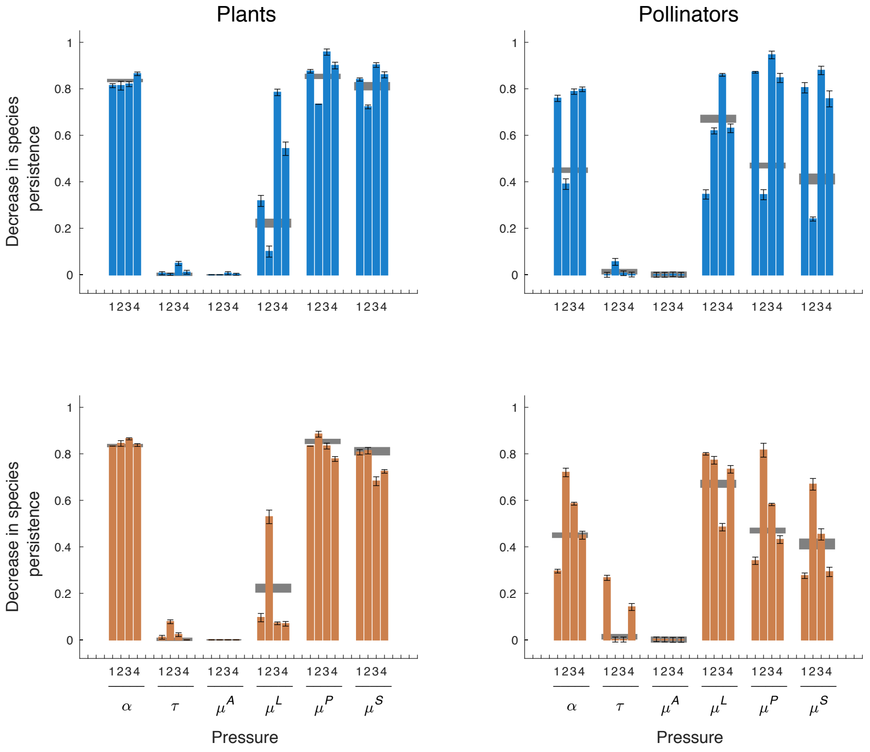


Figure S1: Simulation results for the Zackenberg (1996) network. Left/right plot shows decreases in plant/pollinator species persistence (mean ± 95% C.I.) after exerting different pressures, relative to species persistence without exerting any pressure (see text for details). Experimental pressures, applied one by one, were increased plant competition $\alpha$, decreased visitation rate $\tau$, increased pollinator mortality $\mu^A$, larvae mortality $\mu^L$, plant mortality $\mu^P$ and seed mortality $\mu^S$. Pressure intensities were assigned to species according to their attributes: R: random, D: degree. Ps: phenophase start, Pe, phenophase end, Pd: phenophase duration. In blue, pressure magnitude increased on species of increasing attribute values (low to high D, earlier to later Ps or Pe, and small to large Pd). In orange, pressure magnitude increased on species of decreased attribute values. The smaller bars are shown in front for better view.

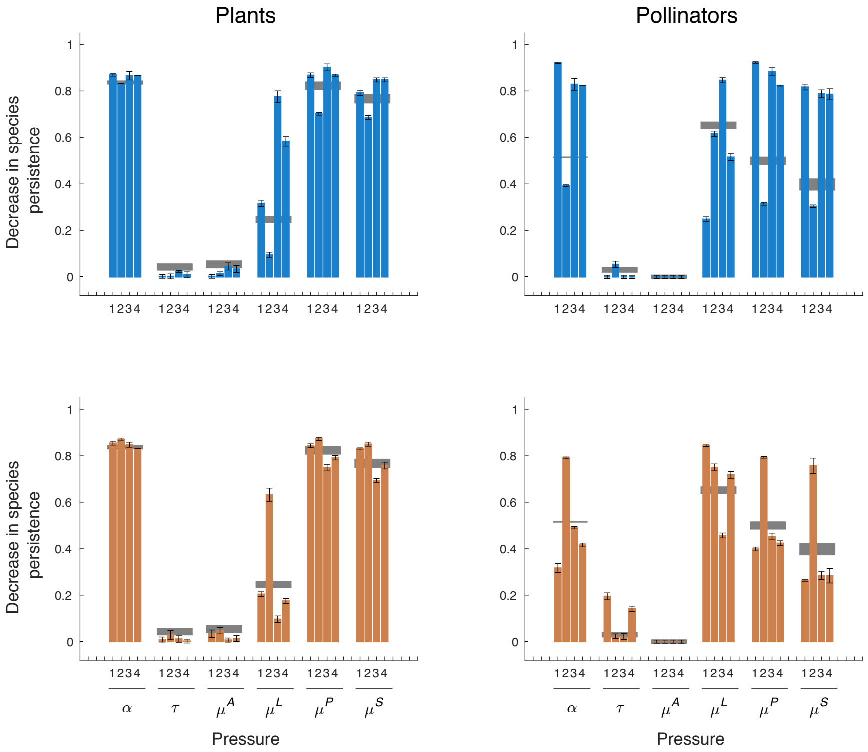


Figure S2: Simulation results for the Zackenberg (1997) network. See Figure S1 for details.

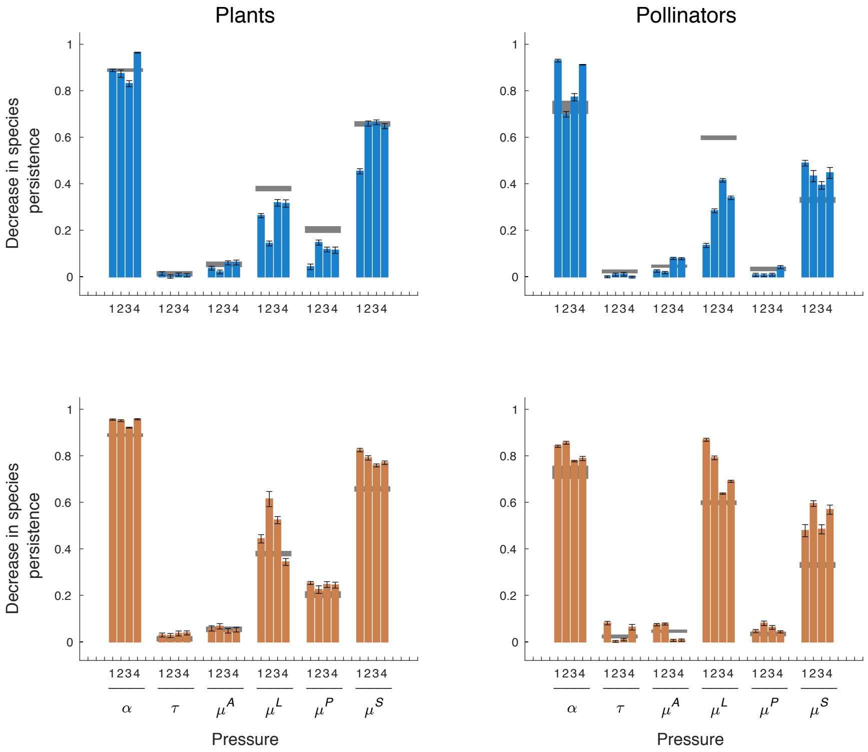


Figure S3: Simulation results for the Athens (1984) network. See Figure S1 for details.

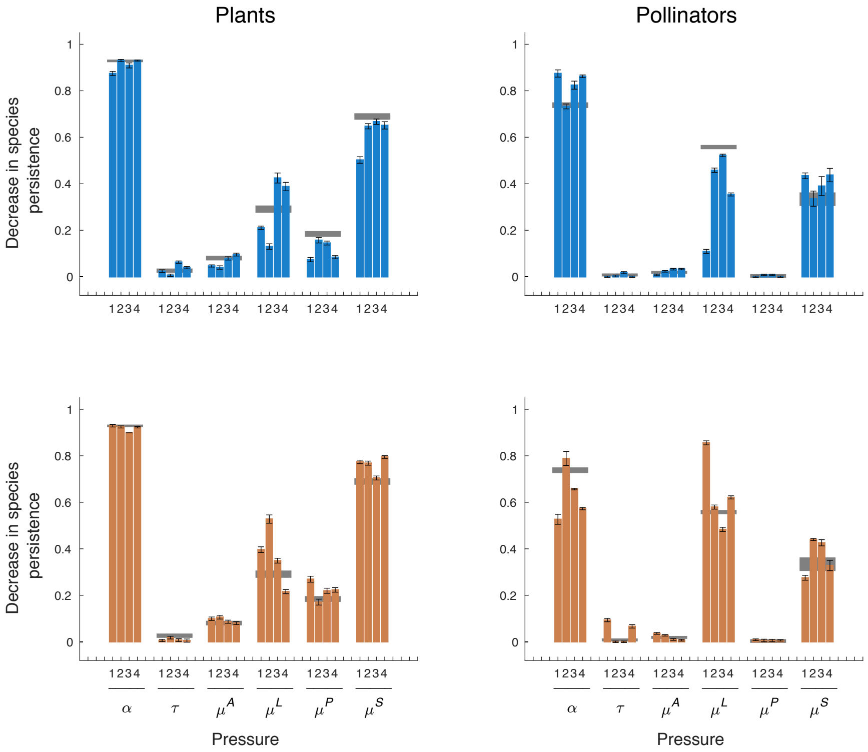


Figure S4: Simulation results for the Athens (1985) network. See Figure S1 for details.

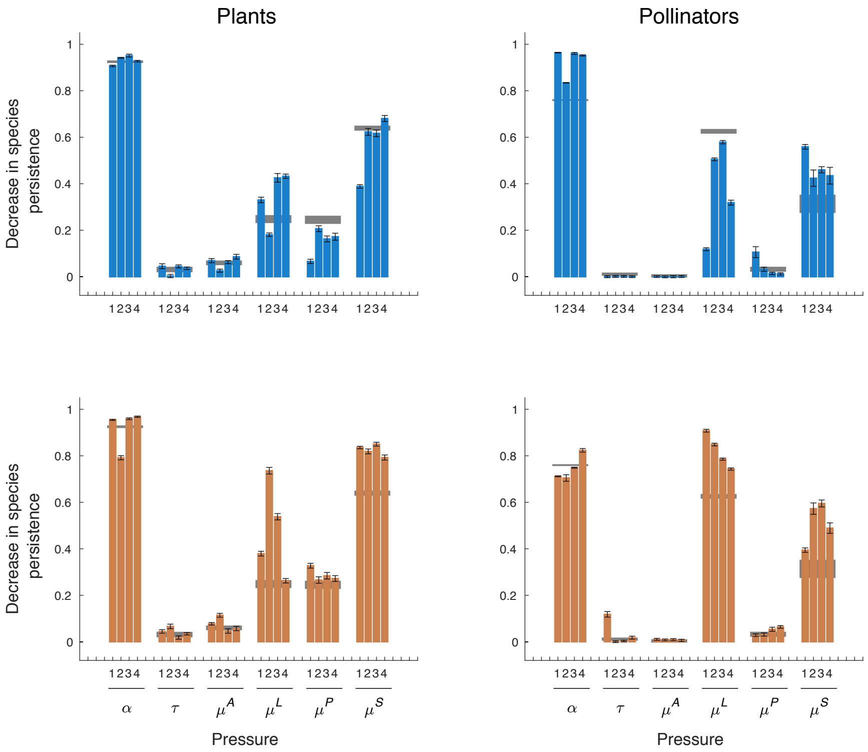


Figure S5: Simulation results for the Athens (1986) network. See Figure S1 for details.

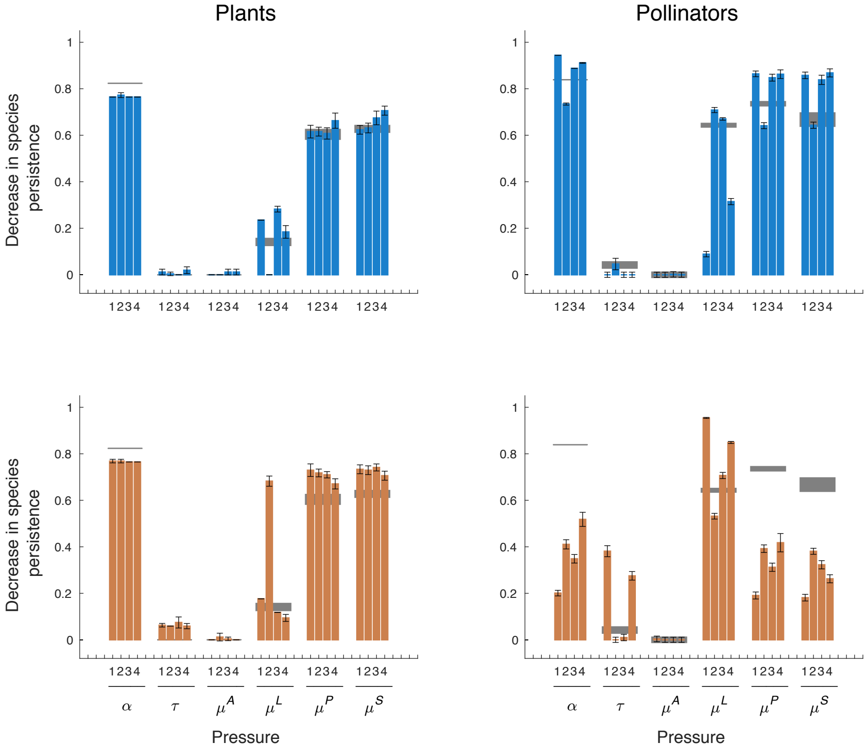


Figure S6: Simulation results for the Nahuel Huapi (1999) network. See Figure S1 for details.

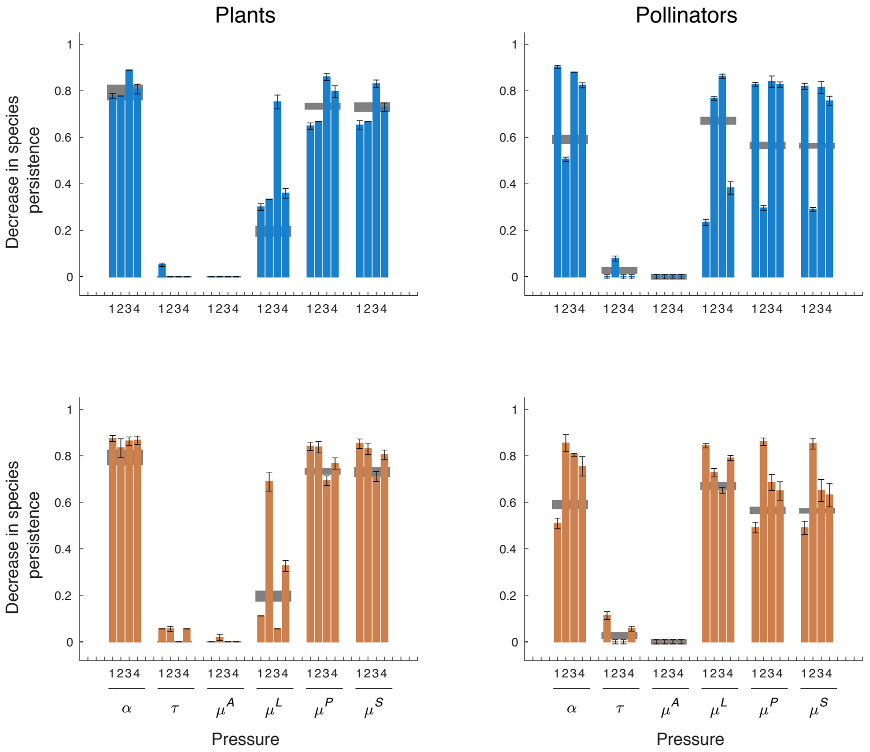


Figure S7: Simulation results for the Villavicencio (2006) network. See Figure S1 for details.

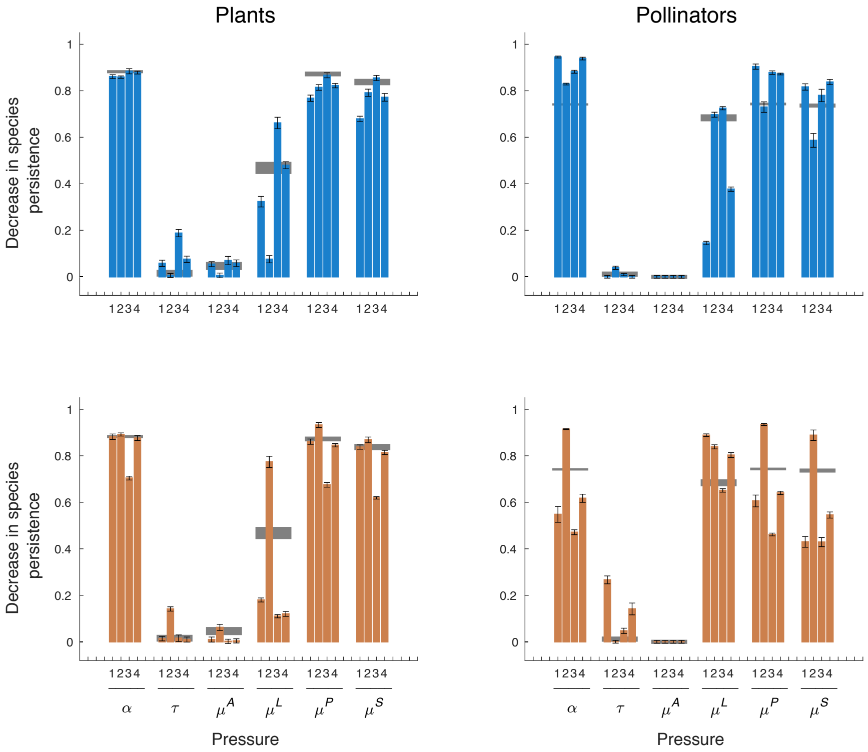


Figure S8: Simulation results for the Villavicencio (2007) network. See Figure S1 for details.

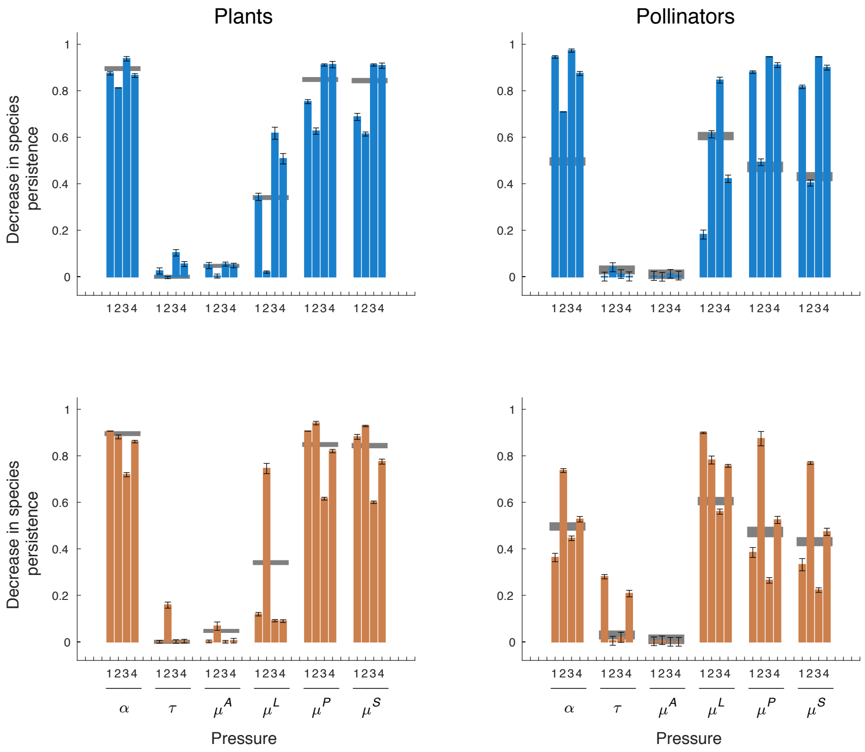


Figure S9: Simulation results for the Villavicencio (2008) network. See Figure S1 for details.

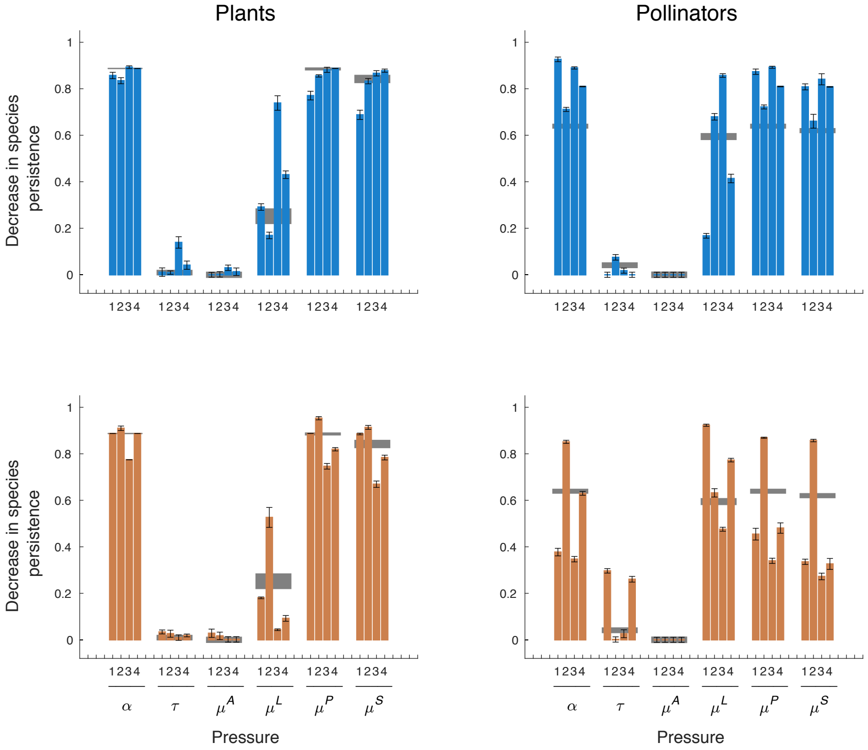


Figure S10: Simulation results for the Villavicencio (2009) network. See Figure S1 for details.

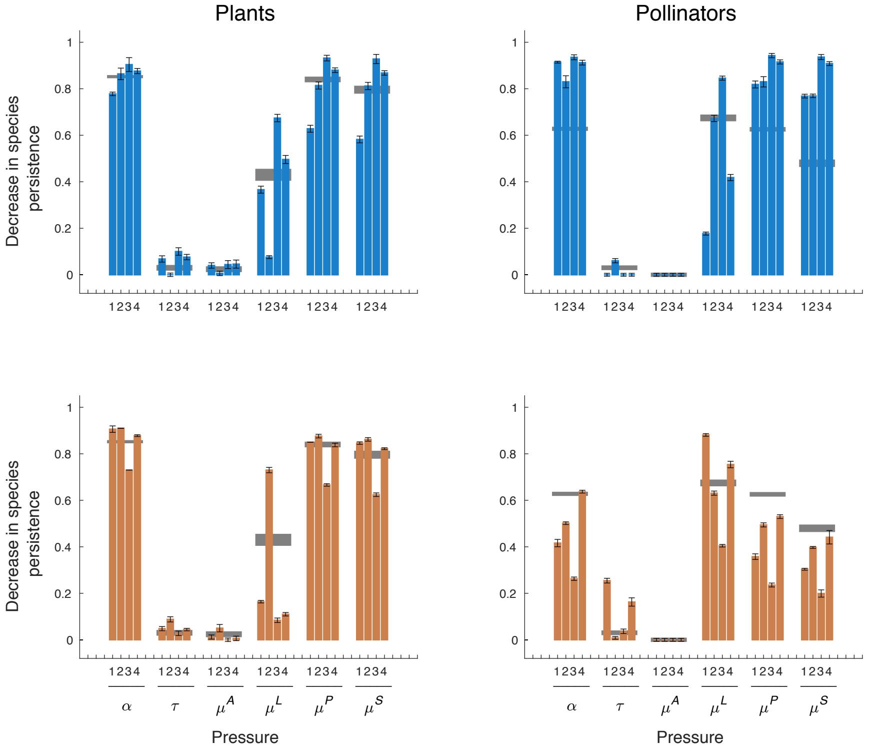

Figure S11: Simulation results for the Villavicencio (2010) network. See Figure S1 for details.

## Supplementary Information S4: NMDS analysis for all tested plant-pollinator networks

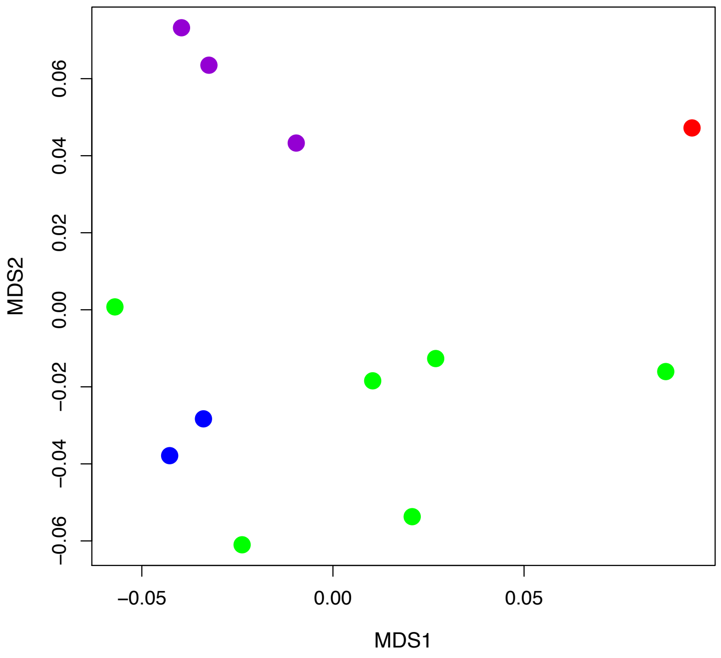


Figure S5: Results of non-metric multidimensional scaling (NMDS) analysis for the 12 networks under study, belonging to four locations: Zackenberg (blue), Nahuel Huapi (red), Athens (violet) and Villavicencio (green).

## Supplementary Information S5: Sensitivity analysis

Here we report results of simulations for the most recent networks at each of the four locations. These simulations were run with the same mean values of parameters and initial conditions, but with a random variation of $10\%, 50\% and 75\%$ around the mean. Results shown in Supplementary Information S3 were obtained with a variation of $25\%$.

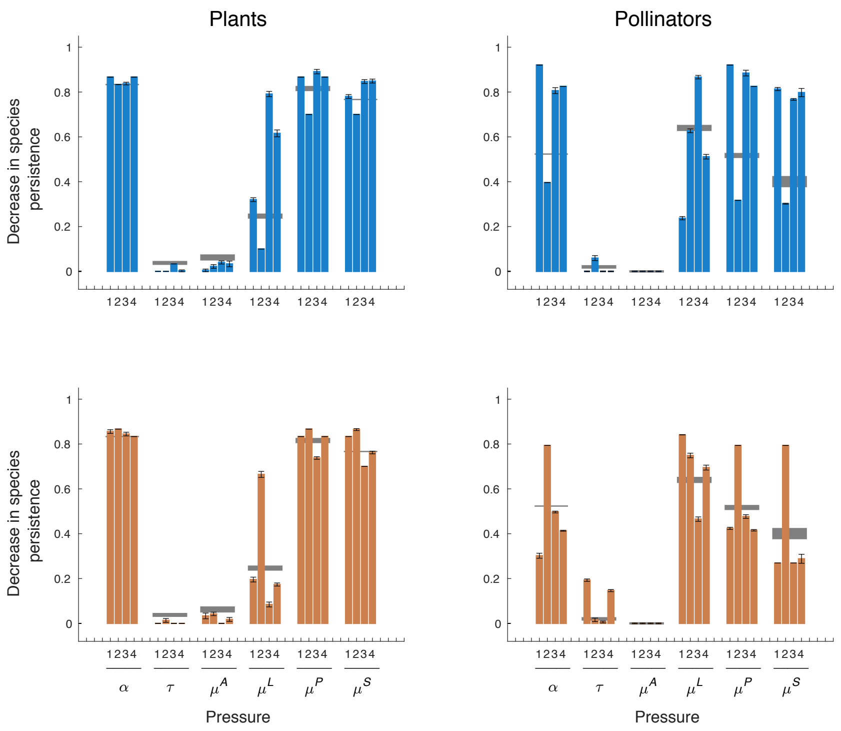


Figure S13: Sensitivity analysis on the Zackenberg (1997) network. Random variation in parameter values and initial conditions of was 10% around the mean.

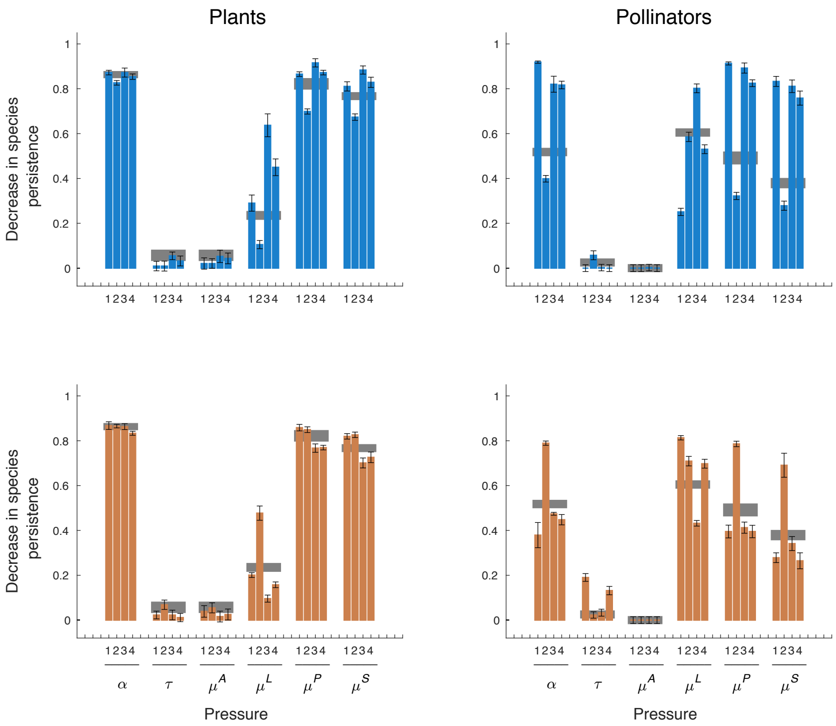


Figure S13: Sensitivity analysis on the Zackenberg (1997) network. Random variation in parameter values and initial conditions of was 50% around the mean.

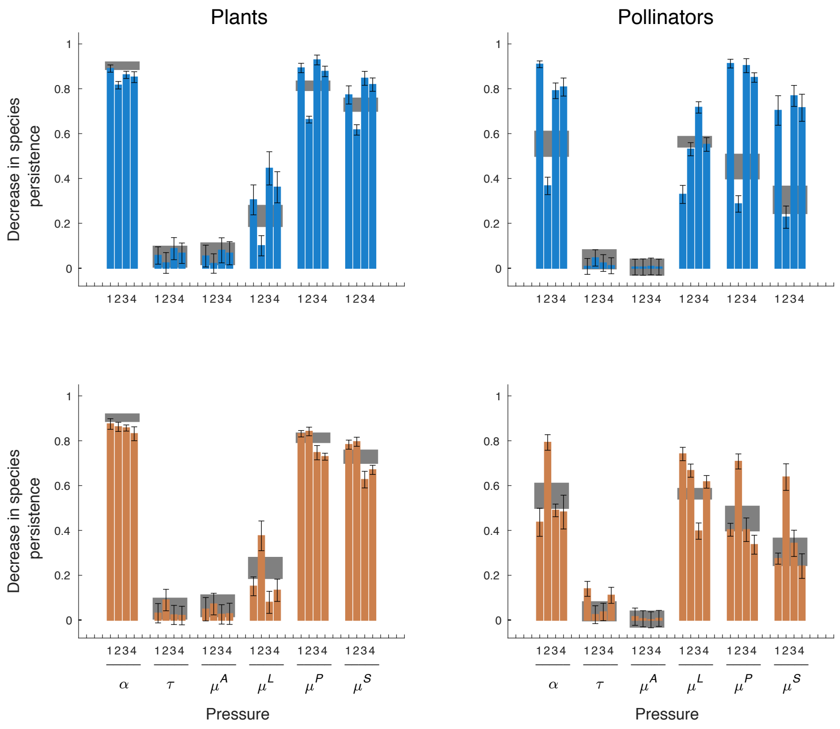


Figure S13: Sensitivity analysis on the Zackenberg (1997) network. Random variation in parameter values and initial conditions of was 75% around the mean.

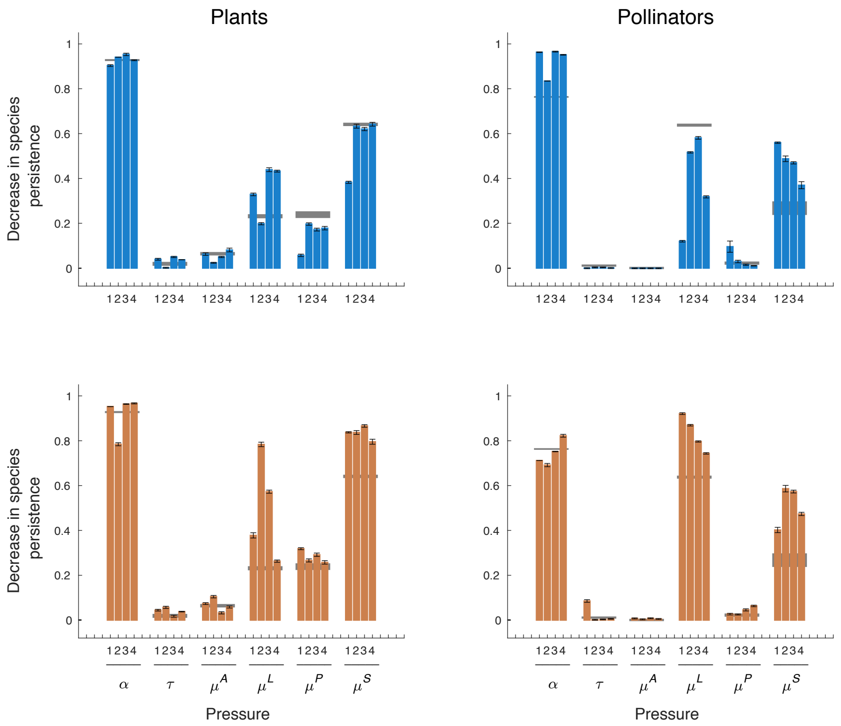


Figure S13: Sensitivity analysis on the Athens (1986) network. Random variation in parameter values and initial conditions of was 10% around the mean.

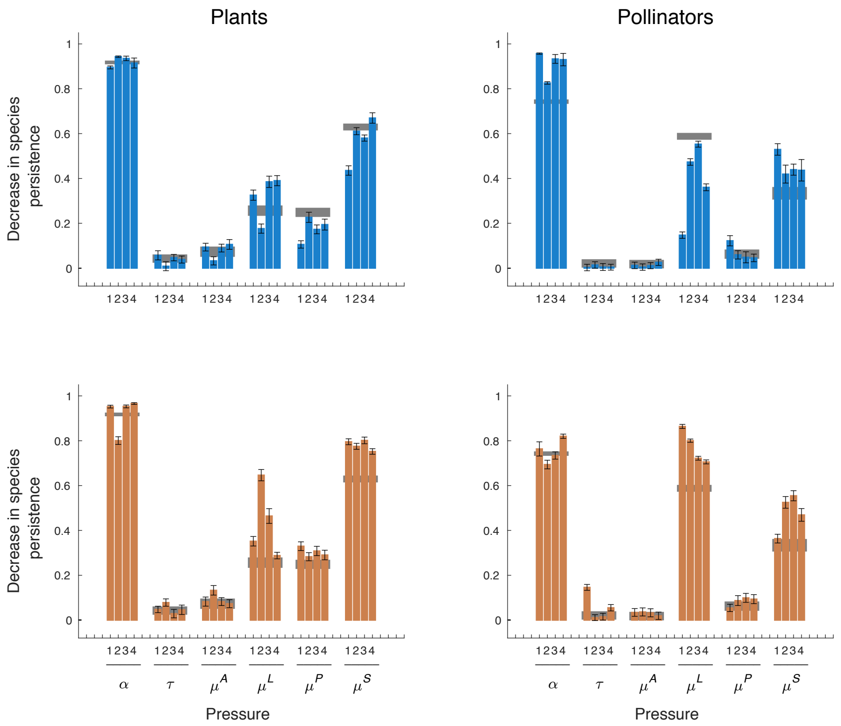


Figure S13: Sensitivity analysis on the Athens (1986) network. Random variation in parameter values and initial conditions of was 50% around the mean.

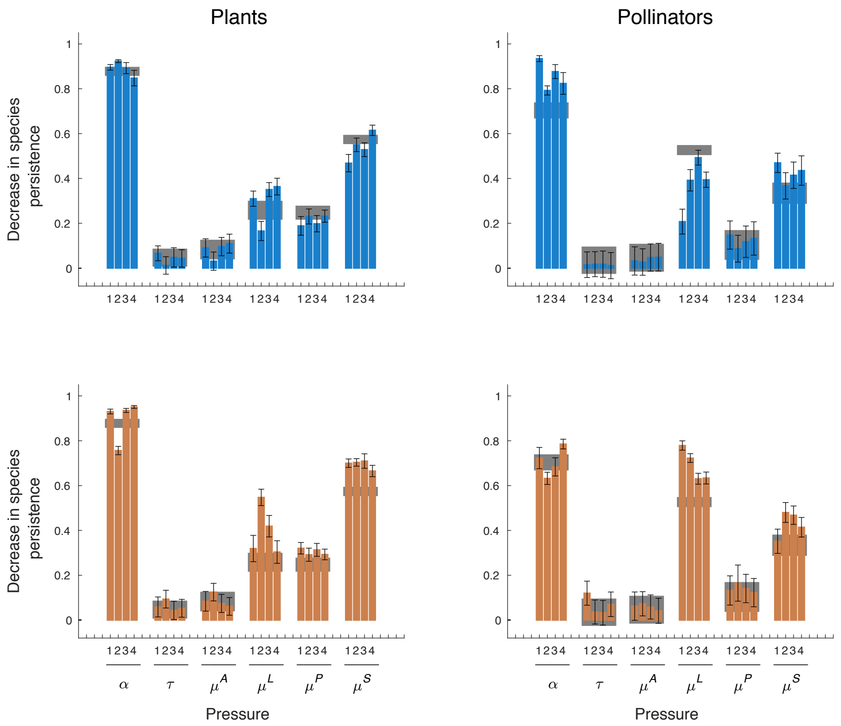


Figure S13: Sensitivity analysis on the Athens (1986) network. Random variation in parameter values and initial conditions of was 75% around the mean.

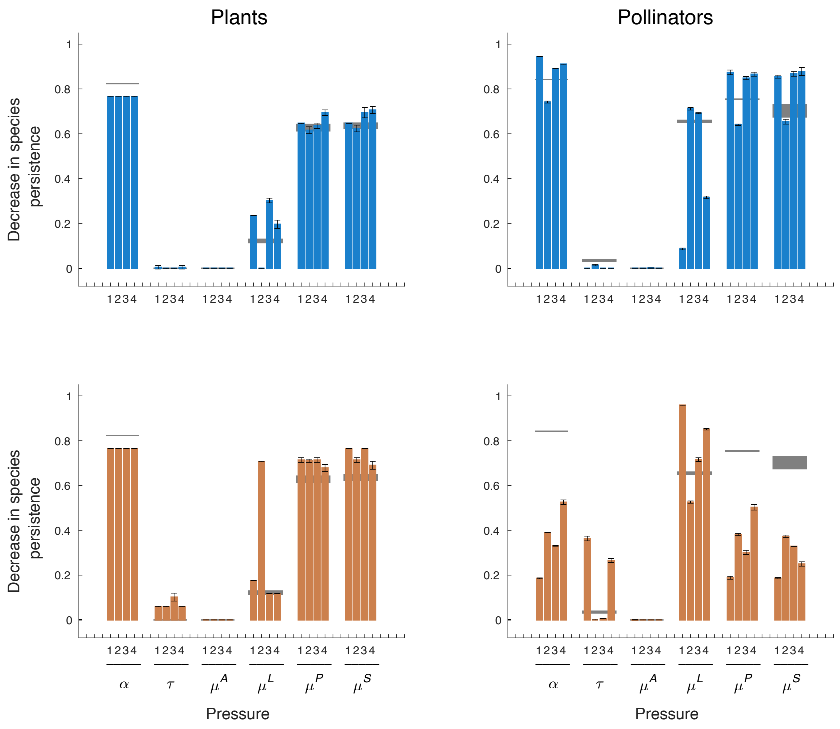


Figure S13: Sensitivity analysis on the Nahuel Huapi (1999) network. Random variation in parameter values and initial conditions of was 10% around the mean.

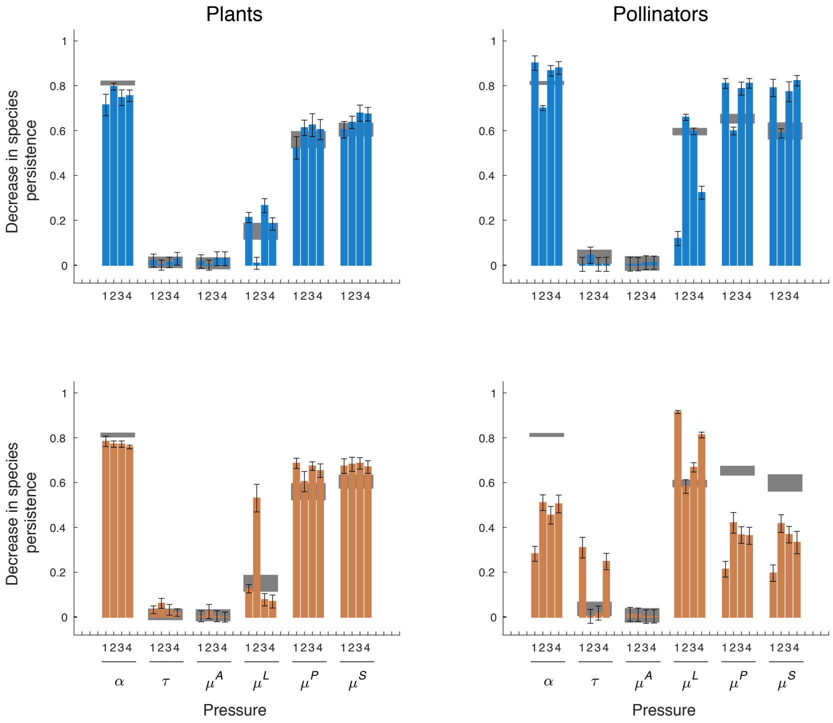


Figure S13: Sensitivity analysis on the Nahuel Huapi (1999) network. Random variation in parameter values and initial conditions of was 50% around the mean.

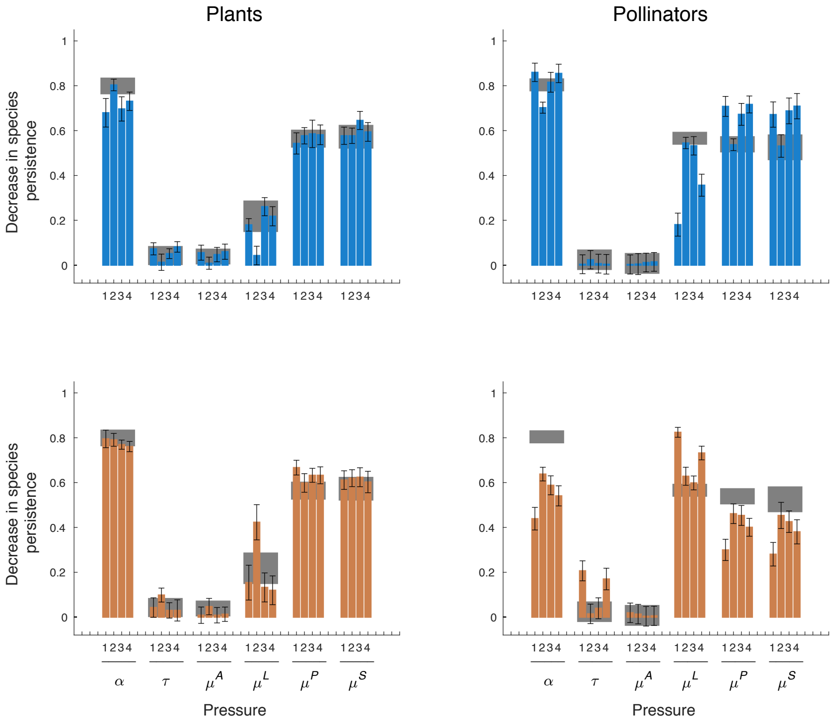


Figure S13: Sensitivity analysis on the Nahuel Huapi (1999) network. Random variation in parameter values and initial conditions of was 75% around the mean.

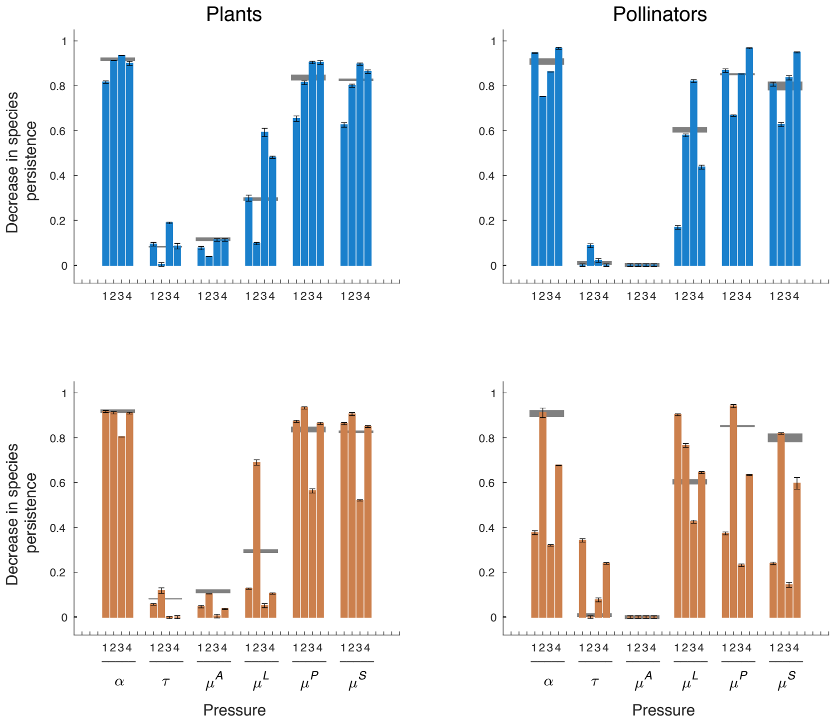


Figure S13: Sensitivity analysis on the Villavicencio (2011) network. Random variation in parameter values and initial conditions of was 10% around the mean.

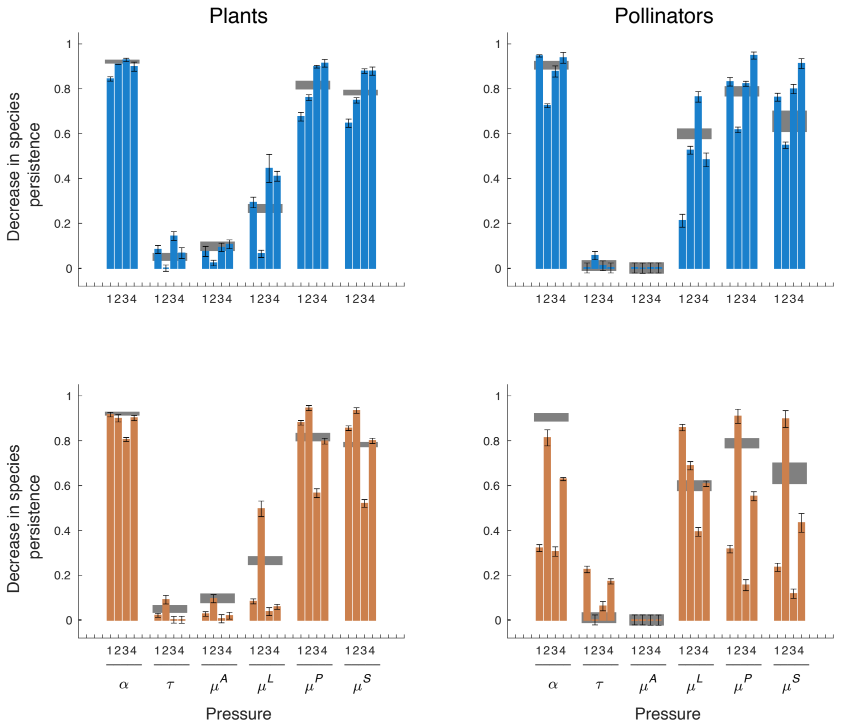


Figure S13: Sensitivity analysis on the Villavicencio (2011) network. Random variation in parameter values and initial conditions of was 50% around the mean.

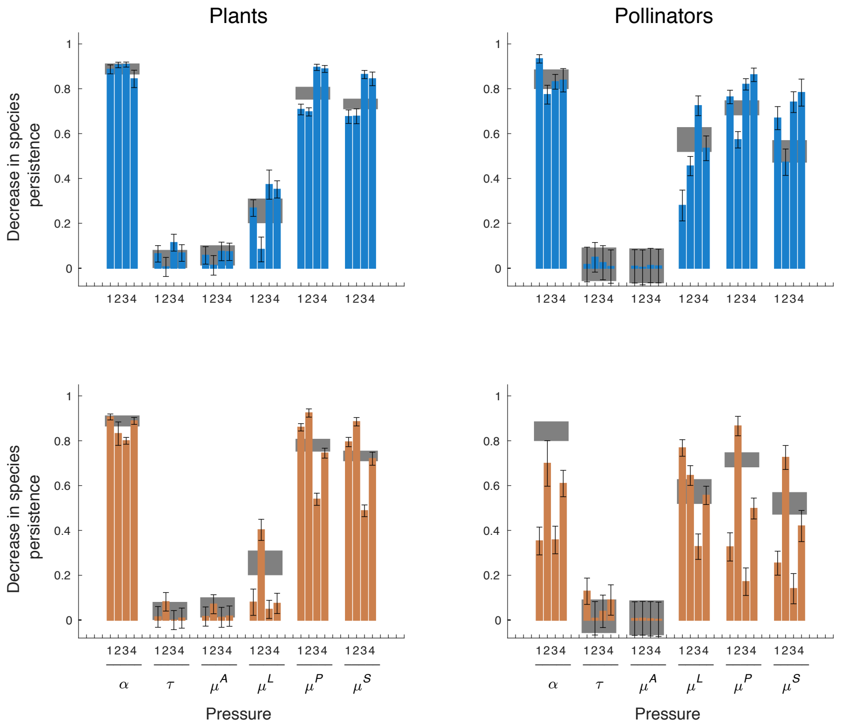


Figure S13: Sensitivity analysis on the Villavicencio (2011) network. Random variation in parameter values and initial conditions of was 75% around the mean.

## Supplementary Information S6: Corrected impact values across networks

**Table 2.** Mean (variance) of corrected impacts on *plant species persistence* of pressures exerted on plant competition ($\alpha$), visitation rate ($\tau$), adult pollinator mortality ($\mu_A$), larval mortality ($\mu_L$), plant mortality ($\mu_P$) and seed mortality ($\mu_S$) of species, according to their degree (D), phenophase start (Ps), phenophase end (Pe), and phenophase duration (Pd). Pressure magnitude increased on species of *higher attribute values* (e.g. high D, later Ps or Pe, and large Pd). Only positive mean values were considered

| | $\alpha$ | $\tau$ | $\mu_A$ | $\mu_L$ | $\mu_P$ | $\mu_S$ |
|---|---|---|---|---|---|---|
| D | - | 0.007 (0.000) | - | 0.033 (0.004) | - | - |
| Ps | - | - | - | - | - | - |
| Pe | - | 0.031 (0.002) | 0.006 (0.000) | 0.276 (0.044) | 0.021 (0.005) | 0.048 (0.002) |
| Pd | - | 0.011 (0.000) | 0.007 (0.000) | 0.143 (0.016) | 0.013 (0.005) | 0.043 (0.001) |

**Table 3.** Mean (variance) of corrected impacts on *pollinator species persistence* of pressures exerted on plant competition ($\alpha$), visitation rate ($\tau$), adult pollinator mortality ($\mu_A$), larval mortality ($\mu_L$), plant mortality ($\mu_P$) and seed mortality ($\mu_S$) of species, according to their degree (D), phenophase start (Ps), phenophase end (Pe), and phenophase duration (Pd). Pressure magnitude increased on species of *higher attribute values* (e.g. low D, earlier Ps or Pe, and small Pd). Only positive mean values were considered

| | $\alpha$ | $\tau$ | $\mu_A$ | $\mu_L$ | $\mu_P$ | $\mu_S$ |
|---|---|---|---|---|---|---|
| D | 0.226 (0.012) | - | - | - | 0.192 (0.028) | 0.243 (0.012) |
| Ps | - | 0.016 (0.001) | - | - | - | - |
| Pe | 0.180 (0.015) | - | 0.004 (0.000) | 0.079 (0.018) | 0.193 (0.036) | 0.236 (0.021) |
| Pd | 0.196 (0.011) | - | 0.003 (0.000) | - | 0.177 (0.023) | 0.236 (0.011) |

**Table 4.** Mean (variance) of corrected impacts on *plant species persistence* of pressures exerted on plant competition ($\alpha$), visitation rate ($\tau$), adult pollinator mortality ($\mu_A$), larval mortality ($\mu_L$), plant mortality ($\mu_P$) and seed mortality ($\mu_S$) of species, according to their degree (D), phenophase start (Ps), phenophase end (Pe), and phenophase duration (Pd). Pressure magnitude increased on species of *lower attribute values* (e.g. high D, later Ps or Pe, and large Pd). Only positive mean values were considered

| | $\alpha$ | $\tau$ | $\mu_A$ | $\mu_L$ | $\mu_P$ | $\mu_S$ |
|---|---|---|---|---|---|---|
| D | 0.002 (0.002) | 0.016 (0.001) | - | - | 0.058 (0.003) | 0.084 (0.003) |
| Ps | - | 0.044 (0.001) | 0.014 (0.000) | 0.390 (0.010) | 0.060 (0.002) | 0.088 (0.001) |
| Pe | - | 0.014 (0.002) | - | - | - | - |
| Pd | - | 0.010 (0.001) | - | - | 0.009 (0.003) | 0.037 (0.006) |

**Table 5.** Mean (variance) of corrected impacts on *pollinator species persistence* of pressures exerted on plant competition ($\alpha$), visitation rate ($\tau$), adult pollinator mortality ($\mu_A$), larval mortality ($\mu_L$), plant mortality ($\mu_P$) and seed mortality ($\mu_S$) of species, according to their degree (D), phenophase start (Ps), phenophase end (Pe), and phenophase duration (Pd). Pressure magnitude increased on species of *lower attribute values* (e.g. low D, earlier Ps or Pe, and small Pd). Only positive mean values were considered

| | $\alpha$ | $\tau$ | $\mu_A$ | $\mu_L$ | $\mu_P$ | $\mu_S$ |
|---|---|---|---|---|---|---|
| D | - | 0.215 (0.011) | 0.005 (0.000) | 0.251 (0.004) | - | - |
| Ps | 0.001 (0.091) | - | 0.003 (0.000) | 0.055 (0.013) | 0.043 (0.082) | 0.102 (0.070) |
| Pe | - | - | - | - | - | - |
| Pd | - | 0.135 (0.007) | - | 0.118 (0.004) | - | - |
